# Research priorities of women at risk for preterm birth: findings and a call to action

**DOI:** 10.1186/s12884-019-2664-1

**Published:** 2020-01-13

**Authors:** Linda S. Franck, Monica R. McLemore, Shanell Williams, Kathryn Millar, Anastasia Y. Gordon, Schyneida Williams, Nakia Woods, Lisa Edwards, Tania Pacheco, Artie Padilla, Fanta Nelson, Larry Rand

**Affiliations:** 1 California Preterm Birth Initiative, University of California San Francisco, San Francisco, CA USA; 2 Department of Family Health Care Nursing, University of California San Francisco, San Francisco, 2 Koret Way, N411F, Box 0606, San Francisco, CA 94143 USA; 3 San Francisco Black Infant Health (formerly), San Francisco, CA USA; 4 Homeless Prenatal Program (formerly), San Francisco, CA USA; 5 Oakland Best Babies Zone (formerly), San Francisco, CA USA; 6 Black Wellness Council, Oakland, CA USA; 7 California State University, Fresno, CA USA; 8 Fresno Every Neighborhood Partnership, Fresno, CA USA; 9 Fresno County Black Infant Health, Fresno, CA USA

**Keywords:** Research priority setting, Research justice, Patient and public involvement, Women of color, Pregnancy, preterm birth, Health disparities, Lived experience

## Abstract

**Background:**

Traditional hierarchical approaches to research give privilege to small groups with decision-making power, without direct input from those with lived experience of illness who bear the burden of disease. A Research Justice framework values the expertise of patients and communities as well as their power in creating knowledge and in decisions about what research is conducted. Preterm birth has persisted at epidemic levels in the United States for decades and disproportionately affects women of color, especially Black women. Women of color have not been included in setting the agenda regarding preterm birth research.

**Methods:**

We used the *Research Priorities of Affected Communities* protocol to elicit and prioritize potential research questions and topics directly from women of color living in three communities that experience disproportionately high rates of preterm birth. Women participated in two focus group sessions, first describing their healthcare experiences and generating lists of uncertainties about their health and/or healthcare during pregnancy. Women then participated in consensus activities to achieve ‘top-priority’ research questions and topic lists. The priority research questions and topics produced by each group were examined within and across the three regions for similarities and differences.

**Results:**

Fifty-four women participated in seven groups (14 sessions) and generated 375 researchable questions, clustered within 22 topics and four overarching themes: Maternal Health and Care Before, During, and After Pregnancy; Newborn Health and Care of the Preterm Baby; Understanding Stress and Interventions to Prevent or Reduce Stress; and Interpersonal and Structural Health Inequities. The questions and topics represent a wide range of research domains, from basic science, translational, clinical, health and social care delivery to policy and economic research. There were many similarities and some unique differences in the questions, topics and priorities across the regions.

**Conclusions:**

These findings can be used to design and fund research addressing unanswered questions that matter most to women at high risk for preterm birth. Investigators and funders are strongly encouraged to incorporate women at the front lines of the preterm birth epidemic in research design and funding decisions, and more broadly, to advance methods to deepen healthcare research partnerships with affected communities.

## Background

Research is one of the last domains of the healthcare enterprise that lacks patient and public involvement (PPI) at every level. However, there is now growing international consensus that healthcare research must be conducted in partnership *with* patients and communities, and not *on* or *for* them. Research conducted in partnership with communities directly affected by the condition under study can improve recruitment and retention, has greater relevance and credibility, and accelerates translation into clinical practice [1–4]. The term “community based participatory research” (CBPR) is often used to describe research projects co-created or driven by community members [5]. Until recently, almost all research, including CBPR, was typically funded by agencies with limited to no requirement of PPI in setting the research strategy or research agenda. Although this is beginning to shift with large national initiatives such as those in the United Kingdom [6, 7], United States (US) [8], and Canada [9], there remain concerns about whether study participants accurately reflect characteristics and views of the people who bear the greates burden of the disease or condition under study and that they have sufficient influence in the decision processes of the funding agencies [10–14]. Therefore, major structural inequalities in the research enterprise persist and perpetuate injustice in healthcare knowledge creation and, as a result, in healthcare delivery [15].

Methods for PPI in research priority setting are relatively nascent. Typical methods of research funders to engage PPI in research strategy include gathering input from representatives from patient advocacy organizations, or inviting patients or patient representatives to stakeholder consultation meetings, sometimes together with professional and industry stakeholders, or conducting surveys [16–20]. One of the most well-developed and widely used methods for PPI in research is the James Lind Alliance (JLA) Priority Setting Partnerships [6] that brings together patients, their caregivers and clinician groups to identify treatment uncertainties (i.e., questions about treatments which cannot be answered by existing research). However, the JLA uses a hierarchical approach that begins with a review of the existing scientific literature to identify gaps in knowledge and because of this may inadvertently privilege traditional medical establishment priorities rather than those of people directly affected by the condition, or those who have been left out of previous research. Moreover, the emphasis on multi-stakeholder forums for reaching consensus, without in-depth work with and sufficient representation of members from the affected communities, may further perpetuate unequal power dynamics in research and further marginalize the unanswered questions and priority research topics of communities that bear the highest burden of health and healthcare disparities.

Recently, we reported on a new method for PPI that is specifically designed to engage members of under-represented communities in research priority setting. The Research Priorities of Affected Communities (RPAC) protocol [13] is designed to elicit research questions and achieve consensus on priority research topics directly from members of communities that experience high burden related to the health condition of interest, and who otherwise would not be included in typical research strategy setting fora. The method is based on a pedagogical framework of Research Justice [15, 21] that seeks to dismantle the traditional hierarchical framework for knowledge generation that privileges certain individuals and institutions with decision-making power over what constitutes ‘legitimate’ knowledge and who can participate in knowledge generation. Research Justice is a more inclusive framework for knowledge generation that seeks to equalize the political power and legitimacy of knowledge generation [22]. In the Research Justice framework, experiential knowledge as well as cultural and spiritual knowledge have equal value with mainstream knowledge.

### The Preterm Birth Epidemic

In 2017, approximately 1 in 10 infants in the US were born too early, before 37 completed weeks of gestation. Preterm birth is associated with significant maternal and infant health risks and worryingly, preterm birth rates are not improving and may be on the increase [23]. The known causes remain poorly understood, explaining only about one-third of preterm births [24]. Primary prevention strategies (e.g., diet and exercise; nutritional supplements; nutritional education; screening for lower genital tract infections) and secondary prevention interventions (e.g., low dose aspirin and L-arginine for pre-eclampsia; treatment of bacterial vaginosis and candidiasis or periodontal disease; progesterone or cervical pessary for short cervix) have shown some efficacy with selected samples in controlled clinical trials [25]. However, population level reduction in preterm birth rates have not been achieved with widespread implementation of these interventions and there has been growing realization that clinical interventions focused on individuals alone are ineffective in addressing the ongoing preterm birth epidemic [26, 27]. Moreover, the US preterm birth rate far exceeds that of other high income countries, and within the US, there are significant health disparities among demographic groups that are not adequately explained by the known clinical causes or access to available treatments, with women of color experiencing higher rates than white women and the highest rates occurring for non-Hispanic Black women [28, 29]. Our thorough search of the literature did not show any prior example of research priority setting with women from communities with high risk for preterm birth.

### Academic-Community Partnership in Preterm Birth Research

The California Preterm Birth Initiative (PTBi-CA) was launched in 2015 to address the disparities in preterm birth rates through place-based community partnered, transdisciplinary, action-oriented research (https://pretermbirthca.ucsf.edu). Early on, we adopted the Research Justice framework [21] and a theory of change that centers the people most affected by preterm birth, positing that breakthroughs in addressing this stubborn epidemic will emerge from work that is fully community partnered, as has been demonstrated with other complex epidemics, such as HIV [30]. In this paper, we report the findings from the application of the Research Justice framework [21] and the RPAC method [13] for the development of a new pregnancy and newborn care research strategy co-created by PTBi-CA researchers and women most affected by preterm birth and other adverse birth outcomes. The findings reported in this paper have directly contributed to our research strategy and can be used to guide others in research agenda setting, strategies, funding and study design to advance knowledge and improve pregnancy and birth outcomes.

## Methods

### Study design

We used a focus group design and quanitative and qualitative analysis to investigate women’s unanswered questions about pregnancy, birth and neonatal care in the context of their own experiences for the purpose of informing and influencing local and national research agendas and funding priorities.

### Setting and Sample

Seven community-based organizations (CBOs) partner agencies serving pregnant of color at high medical and/or social risk for preterm birth in Fresno, Oakland, and San Francisco were invited to participate as partners in the research. We chose the settings because of the high preterm birth rates for women of color in those communities [31]. The CBO partners provided prenatal, childbirth, post-partum or parenting classes, or other individual or community skill-building or support services for pregnant women and families with young children. Additionally, the CBOs needed to have the capacity to host the two focus group sessions, provide a staff member for co-facilitation of the sessions, provide refreshments and childcare, organize transportation, and assist with participant recruitment. CBO partners were compensated for these services. CBO partner staff posted fliers about the project in their locations and personally invited clients who met the inclusion criteria to participate using a standard script.

Pregnant or parenting women were eligible to participate if they self-identified as Black, Hispanic, Latina, or mixed-race, age 18 or older, English-speaking or monolingual Spanish-speaking (Fresno only), received services from one of the partner CBOs, and had a current or previous pregnancy. Participants received an information sheet about the study, signed audio-recording and photography consent and received $50 for participating in each of the 2-h focus group sessions.

The study was reviewed and deemed exempt from human subjects protection procedures by the University of California, San Francisco Committee on Human Research # 15–15,698.

### Procedures

The focus group interview guides were developed in partnership with the San Francisco CBOs in accordance with the RPAC protocol [13]. The same interview guide was used at all sites, with minor adaptations to fit the local context (e.g., different introduction and grounding exercises). Women participated in two audio-recorded focus group sessions, 2 h in length, 4 to 6 weeks apart, facilitated by the first and second authors and a CBO co-facilitator. In the first session, participants described their healthcare experiences and generated potentially researchable questions based on uncertainties or unanswered questions about their health and/or healthcare during current or recent pregnancies. In all groups, the participants moved rapidly from hearing their statements of experience reframed as possible questions, to generating questions themselves in their own words (see reference 13 for a detailed facilitator script; for a video demonstration of the process see: https://www.jove.com/video/56220/a-novel-method-for-involving-women-color-at-high-risk-for-preterm). Facilitators refrained from specific discussion of the available evidence for the questions posed by the participants and focused on thoroughly eliciting all of the participant’s unanswered questions. If participants asked for factual information (e.g., statistics of the prevalence of a specific condition), these were placed in a ‘parking lot’ to be investigated by the research team and reported on in the second session.

Between the sessions, the research team transcribed the questions from the flip chart notes and transcript and thematically organized them under research topic categories. The topic categories were kept consistent across groups, with new ones added only for new topics. Additionally, after the first focus group, the research team removed any questions from the question list if there were definitive answers to these questions supported by national professional guidelines or rigorous systematic reviews of published research. They also compiled responses to factual questions posed by the participants.

In the second session, women reviewed the cumulative list of the unanswered questions they generated in the first sessions, confirmed that the topic categories were appropriate (or made adjustments), and generated additional questions. Facilitators provided answers to factual questions posed in session 1, and noted if any questions had been removed from the question list because of definitive research addressing those questions, and provided those references. The women then participated in multiple rounds of individual and group voting and discussion to achieve consensus on the order of priority of the research topic categories and on a ‘top-priority’ research question list, selected and agreed from the full list of research questions [13]. The second session closed with women sharing their views about participation in this process and their reflections on what having answers to their priority questions might mean to the future healthcare and health outcomes of their communities.

The focus groups with monolingual Spanish-speaking (or Spanish-preferred) women were conducted in Spanish with three native Spanish speakers serving as facilitators. Additionally, all materials for those sessions, including the transcript were translated and reviewed by the team who conducted the focus groups, translated into English, reviewed by a Spanish speaking medical interpreter and a Spanish-speaking researcher prior to analysis.

### Analysis

The priority research questions and topics produced by each group were examined within and across regions for similarities and differences using weighted rankings and thematic analysis [32]. First, the priority topics from all seven groups were analyzed according to the rank they were assigned by each group. For each topic, a mean rank score was derived by summing the topic rank score across all of the groups and dividing by the number of groups for which that topic was deemed a priority topic. Then, to account for the frequency each topic appeared across the sites, the mean rank score was weighted by the total number of sites that prioritized the topic. The topics were then ordered by lowest to highest weighted mean rank score across all regions.

The priority topics and questions were also examined qualitatively using thematic analysis [32] to determine themes and subthemes by region and across regions. The first and second authors listened to the recordings, reviewed the transcripts for accuracy and conducted first level coding of topics. A larger subgroup of authors developed preliminary themes and subthemes for topics and questions across regions. The full author group reviewed the themes. Disagreements were resolved by discussion and an iterative process was used to refine the themes and choose the quotes to be included as exemplars.

## Results

A total of 54 women participated in 7 research prioritization groups conducted in Oakland (*n* = 2), San Francisco (*n* = 2), and Fresno (*n* = 3) between April 2015 to June 2017. Each group participated in two focus group sessions (*n* = 14 sessions). One research prioritization group (two sessions) was conducted in Spanish in Fresno. Three participants were unable to participate in the second sessions because they or their children were ill that day.

Participant age ranged from 20 to 44 year. The participants had given birth to a total of 122 children, of which 45 were born preterm (37%). Eleven of the children (9%) had died prior to the focus groups. The self-identified race/ethnicity of the participants by region is shown in Table 1. Many of the participants had been told by their pregnancy care providers that they were ‘high risk’ and required frequent healthcare visits during their pregnancies. Some participants were not formally labeled by their providers, but perceived themselves to be at ‘high risk’, while others felt they had ‘normal’ pregnancies and yet they and or their infants experienced adverse events during pregnancy or after birth.

**Table 1:** Self-identified race/ethnicity of focus group participants by region

Region	Number of Participants in Focus Group 1	Number of Sites	Number of Participants by Self-identified Race/Ethnicity		
			Black	Hispanic	Mixed
Fresno	26	3	8	18	0
Oakland	14	2	9	3	2
San Francisco	14	2	13	0	1
Total	54	7	30	21	3

During the priority ranking activities, participants often commented that all of the topics and questions were important, but they were able to complete the tasks by staying focused on the goal of prioritizing what research was most urgent to do immediately or very soon. At the end of the second session, all 51 women who attended expressed a similar view that they found that the group discussion with other women like themselves ‘healing’. Many of them shared that it was the first time they had been able to speak about their pregnancy, birth and newborn caregiving experience with others like themselves and they found this was a highly-valued, and often emotional, experience.

### Priorities across regions

Across the seven groups, a total of 375 researchable questions were generated by the participants, representing 22 distinct research topic areas. Only a few questions were removed from the participant initial generated lists in session 1 because they could be directly answered (e.g., What is the rate of preterm birth for African Americans in my county? [33]; How effective are IUDs in preventing pregnancy? [34]; How do maternity leave policies vary by country? [35]), or because there were well-established national guidelines or systematic reviews (e.g., Are homeless women at greater risk for poor birth outcomes [36]; Do preterm infants born closer to term have later health problems? [37]).

Considering the relatedness of many of the priority research topics and specific questions, we identified four overarching research themes, that encompasses the research questions across all groups and region: Maternal Health and Care Before, During, and After Pregnancy; Newborn Health and Problems Related to Prematurity; Understanding Stress and Interventions to Prevent or Reduce Stress; and Research to Address Interpersonal and Structural Health Inequities. The overarching themes, top priority topics across all counties and sample questions are shown in Table 2. See Appendix 1 and Appendix 2 for a list of for each group’s top priority topics and top-ranked potential research questions, respectively. A full list of the research questions by topic and by region are available at: https://pretermbirthca.ucsf.edu/developing-research-strategy-partnership-communities-affected-preterm-birth.

**Table 2:** Overarching Themes, Priority Research Topics by Rank and Example Priority Potential Research Questions

Overarching Theme	Priority Research Topics (Rank Importance Across All Regions)	Example Priority Potential Questions (Region)
Maternal Health and Care Before, During, and After Pregnancy	• Medications, Procedures and Tests During Pregnancy [1] • Mother’s Health Before and During Pregnancy [3] • Care Provision During Pregnancy and Birth [4] • Care After Birth [5] • Decision Making During Pregnancy [11] • Preconception, Contraception and Sexuality [12] • Mental Health Support [13] • Maternal Nutrition [15]	Why don’t we have alternative options for support during delivery? (For example, doula, midwife) (Oakland)
Newborn Health and Care of the Preterm Baby	• Newborn Health and Problems Related to Prematurity [6] • Infant Nutrition, Feeding and Medications [14]	Is it harmful to babies to be separated from mom (family) even if they are sick? (Fresno)
Understanding Stress and Interventions to Prevent or Reduce Stress	• Stress and the Benefits of Social Support [2] • Education and Empowerment of Birthing Women [7] • Support for Fathers During and After Pregnancy [16] • The Impact of Men on Pregnancy Outcomes [17] • Role of Family and Friends in Caring for Families [19]	Why aren’t there more widely available education materials or videos, etc. to help women recognize their own stress? (Fresno)
Research to Address Interpersonal and Structural Health Inequities	• Health Care Providers [6] • Communication and Cultural Sensitivity [8] • Hospital and Health System Practices [9] • Workplace and Insurance Issues [10] • Innovations in Care Models [18] • Pharmaceutical Company Involvement in Birth [20]	What are the most effective ways to improve patient-provider communication, particularly when patients perceive insensitive and rude comments from health care workers? (San Francisco)

For further details by group and region, refer to Appendices 1–2 and website: https://pretermbirthca.ucsf.edu/developing-research-strategy-partnership-communities-affected-preterm-birth

Examining the topic priority lists across groups, three research topics were prioritized by all groups, and ranked highly for the groups: Medications, Procedures and Tests During Pregnancy; Care Provision During Pregnancy and Birth; and Healthcare Providers. Three topics were listed by six of the seven groups: Communication and Cultural Sensitivity; Workplace and Insurance Issues; and Newborn Health and Care and Problems Related to Prematurity; but the ranking of these priority topics was more variable across the groups. An additional 5 priority topics were common across the 5 groups in Oakland and Fresno only: Stress and the Benefits of Social Support; Education and Empowerment of Birthing Women; Mother’s Health Before and During Pregnancy; Hospital and Health System Practices; and Preconception, Contraception and Sexuality. There were several topics unique to Fresno and Oakland Regions. Fresno women were interested in more research on the involvement of pharmaceutical companies in birth and Oakland women were interested in more research on innovative care models. Unanswered questions within each of the overarching research themes and subthemes are described below, highlighting similarities and differences across the regions and with illustrative questions.

### Maternal health and care provision before, during, and after pregnancy

Across all regions, participants generated the largest number of priority questions for research focused on health and healthcare services for women before, during >and after pregnancy. Specifically, participants had questions about maternal health conditions that might affect pregnancy; utility, safety, and effects of procedures, tests and medications during pregnancy as well as about the ways in which care is delivered during labor and delivery. They also had many questions about patient-provider interactions and health systems, focused on decision-making process and observed differences or disparities in approach.

Maternal health before pregnancy: Questions regarding contraception, birth spacing, and family planning were common across the regions. In Fresno the questions focused on optimal birth spacing specifically after having a preterm birth or other complications, and if recommendations differed for women who hadn’t had a preterm birth or adverse pregnancy outcome. Participants in Oakland wondered about specific types of contraceptive methods, such as intrauterine devices or tubal ligation and they also wanted more research on why some women still get pregnant even while using contraception and what women can do to prepare their bodies for pregnancy. Participants in Fresno asked: Does the recommended pregnancy interval differ/change for pregnancy conditions or birth outcomes? Participants in San Francisco did not propose research questions on pre-pregnancy health, contraception or sexuality.

Procedures, tests and medications during pregnancy: Across all regions, participants had questions about the utility and harm of tests and procedures during pregnancy and labor. Participants often commented that the tests, exams and procedures that were performed on them seemed to be dependent on provider and based on individual bias or preference rather than on facts, and wondered if there could be an agreed upon standard for laboratory tests, exams and procedures in pregnancy. These questions were often quite pointed, such as: “Why do medical providers hurt us so much when performing physical exams on us during pregnancy and when we come in for our ultrasounds?” In Fresno, participants posed questions about the necessity and side effects of a wide range of tests and treatments during pregnancy, including the utility and potential harm (e.g., bleeding) of vaginal exams. Fresno and San Francisco participants had questions about the safety and side effects of tests and medications for the fetus. They wanted to know why more providers did not recommend natural remedies for problems during pregnancy. Participants in Oakland wanted more research on the necessity and utility of non-stress testing and the effects of non-adherence to recommendations for use of prenatal vitamins and they also wanted more information about the accuracy of genetic testing and when it should be done, how to make guidelines more transparent, and what specifically the guidelines are for nurses in educating patients about genetic testing.

Causes and risks for preterm birth: In Fresno and San Francisco, participants posed questions regarding the causes of preterm birth, and in particularly why it occurs for some pregnancies and not others (seemingly inconsistently related to known risks). Participants in all regions also wanted to know the causes of health problems in pregnancy and effects on pregnancy and fetus. Participants in Fresno and San Francisco asked why bleeding happens in pregnancy, what level of bleeding is safe, or if any bleeding meant a risk of miscarriage. Participants in San Francisco and Oakland wondered if maternal age affects test results and if maternal age is a risk factor for problems in pregnancy, including preterm birth and poor breastmilk production. Participants in Oakland generated several questions about diabetes and it’s effects on preterm birth, while participants in Fresno focused their questions on other serious maternal conditions such as polycystic ovarian syndrome; hemolysis, elevated liver enzyme levels, and low platelet (HELLP) syndrome; placental abruption; and infection.

Labor and delivery care practices: Participants in Oakland and Fresno wondered why epidurals don’t always work and cause back problems, or if they are even necessary and how decisions are made to intervene during labor. In Oakland and Fresno, participants wondered how long is safe to labor before a cesarean section is performed and how the decision to perform a cesarean section is made. In Oakland and San Francisco, participants wanted more research on birth plans and how to ensure the health team follow women’s birth plans. In Oakland and San Francisco, many participants wondered if it was safe to keep the fetus in the womb after their water had broken and for how long before infection was a risk. In Oakland, participants focused on research to identify active labor and what to do when labor doesn’t progress. They also wanted more research on how the relationship between laboring women and the care team could be improved, for example: “Why don’t we have alternative options for support during delivery, such as a doula or midwife?”

Patient-provider interactions: The roles of and interactions between patients and their providers was an important subtheme of care provision. Participants in Oakland wondered “What can we do to help our providers to help us?” In Fresno, participants asked “why don’t providers trust patients who know their own body?” Participants in all regions also inquired about inconsistencies in roles and responsibilities of their health care providers, with most of these questions coming from participants in Oakland. They also wanted more research on quality of care differences between providers and on causes and interventions to prevent provider delays in ordering needed diagnostic testing (e.g., for a woman who had experienced multiple miscarriages).

Health system questions: Participants in all regions posed questions about health systems, such as how effective the current prenatal visit schedule is and if there is a way to better streamline pregnancy care, especially for women with chronic stress and acute illness, so women get “what they need, when they need it, regardless of insurance.” Participants in Fresno desired visits and tests closer to home and wondered if team-based models of care are effective. They pointedly asked: “Why aren’t the staff at doctors’ offices diverse when California is a diverse state?” In San Francisco, participants wanted to know how monitoring during pregnancy can be streamlined and if and how women can opt out when the burden of monitoring becomes too much. In Oakland and San Francisco, participants wanted more research on more effective and woman-centered care. Participants in San Francisco and Oakland, in particular, wanted more research on how the health care and social service systems could provide better care and support in the postpartum period. Participants in Oakland posed questions on the best way to heal and rest after giving birth and those in Fresno posed questions about why postpartum depression occurs in some women.

### Newborn health and problems related to prematurity

Newborn health, and particularly that of the preterm baby, was a shared research priority across all regions. Participants generated many research questions about the most appropriate care for infants in the neonatal intensive care unit (NICU) setting. In Fresno, participants had questions about accuracy of anthropometric measures and participants in Fresno and Oakland wanted research to eliminate the barriers to contact with babies when there is an NICU admission or the mother has a cesarean section and support for breastfeeding mothers of preterm infants, asking: “Why can’t accommodations be made to keep moms and babies together after cesarean section?” (Oakland) and “Could train-the-trainer experienced moms be used more effectively for breastfeeding support?” (San Francisco). Additional questions regarding newborn health and prematurity were noted in each of the three regions related to participants’ own experiences. In Oakland, participants had specific questions about breastfeeding. In Fresno, participants wanted to know more about newborn medications, including effects of medications and clinical decision-making about medication use.

Participants were also concerned with the clinical and developmental outcomes for babies born preterm and predicting long-term developmental outcomes: “How can we tell if they will catch up?” (Oakland group). Participants in Fresno and Oakland wanted more research to understand risks and complications of prematurity on infant health. In Fresno, participants wanted more research on care at home after preterm birth, such as what parents could do to support their preterm infant’s development. Participants in San Francisco and Fresno also wanted more research on the best methods for monitoring and follow-up care for preterm babies. In Fresno, home monitoring and follow-up were important areas for further research and in San Francisco, participants wanted research on whether experienced parents of former preterm infants could be used as support for new mothers.

### Understanding stress and interventions to prevent or reduce stress

All groups had a strong interest in research to better understand the role of stress and preterm birth and other adverse outcomes. For eample: “What are the major stressors during pregnancy and the impact these have on preterm birth?” (San Francisco). Participants in all regions prioritized research related to discovering more about methods to increase support and reduce stress that could adversely affect pregnancy and newborn health. Participants in San Francisco generated the most questions related to this theme and participants from Oakland generated the fewest. San Francisco and Fresno participants asked for research to determine which social supports improve patient satisfaction in birth experiences or impact clinical birth outcomes. In Fresno, women asked: “What can be done to improve assessment of stress - with providers or self-assessment?” In San Francisco, participants wanted research on the best strategies for preparation for parents at risk preterm birth and supports for stressed parents of preterm infants. They also had questions regarding health and social care system stressors during pregnancy, including drug testing and Child Protective Services (CPS) involvement, and if those led to increased risk for preterm birth.

In San Francisco, participants were concerned with the stress related to single parenthood while pregnant or giving birth. In Fresno, unique questions were generated about mental health and stress prevention. They also wanted more research on stress prevention and wanted to know why stress prevention resources were not better coordinated and streamlined and “Why isn’t there more funding for education to prevent stress and why isn’t it more widely publicized?”

### Research to address interpersonal and structural healthcare inequities

Groups in all regions posed questions about racial disparities and healthcare inequities as high priority for research. They asked very pointed questions about whether diagnosis, treatment and care decisions were made by clinical providers or healthcare payers based on race or country of origin and why different race and ethnic groups receive different types of care. For example, “Why does Hospital X have an Asian floor, Chinese module and Spanish module, but nothing for people like me - why isn’t there an African American floor? In San Francisco and Fresno, participants prioritized questions exploring both the relationship between insurance type and healthcare disparities, having observed that insurance type sometimes resulted in differing levels of healthcare quality and access to services. Participants in San Francisco and Fresno had questions about differences in healthcare provision between the U.S. and other countries. Participants in San Francisco wondered why other countries provided paternal leave, whereas the U.S. does not, and participants in Fresno wondered if pregnancy-related care differed between countries.

Another common subtheme regarding inequities in healthcare was the interaction between healthcare providers and patients, specifically around communication, respect, cultural humility, and the role of trainees in providing care. They also were interested in research about power dynamics and providers’ decision-making processes. They had specific questions related regarding nursing interactions with patients: “Why are nurses disrespectful and/or do not believe patients?” (San Francisco) and were also interested in research on profit motives of physicians, hospitals and pharmaceutical companies, asking: “Why do obstetricians and gynecologists see us as a business?” (Fresno).

## Discussion

This study represents the first in-depth exploration of the unanswered questions and research priorities of women of color from communities experiencing high rates of preterm birth and other adverse birth outcomes. We found that women had many unanswered questions about the causes, prevention, consequences and treatment for health problems related to pregnancy and for newborns, with many related to preterm birth. All of the participants in all of the groups were readily able to generate specific potential research questions and broader research topics without any prior research or clinical expertise and with minimal instruction during the sessions. Together, they generated 375 questions, clustered within 22 topics and four overarching themes: Maternal Health and Care Before, During, and After Pregnancy; Newborn Health and Care of the Preterm Baby; Understanding Stress and Interventions to Prevent or Reduce Stress; and Interpersonal and Structural Health Inequities. There were some differences in the top priority questions and topics as well as in the specific questions across regions. This appears to be related to the different health care contexts and lived experiences of the participants. Overall, however, there were more similarities than differences and in priorities across all groups. The questions and topics represent a wide range of research domains, from basic science, translational, clinical, health and social care delivery to policy and economic research. A search of the current research literature demonstrates many areas of conceptual alignment between the priorities of women with lived experience of preterm birth risk and researchers in this field [38]. The findings share some similarities with previous findings from the James Lind Alliance preterm birth priority setting partnership in the United Kingdom [39]. However, the priorities of women of color were broader and included greater focus on the social determinants of health and a call for more research on interpersonal and structural health inequities.

Moreover, many of the topics and questions of great interest to the participants in this project (e.g., interpersonal and structural healthcare inequities) do not receive the same level of investment as those topics of interest to researchers and institutions (e.g., placental biology). This has been the case with other conditions (e.g., cerebral palsy disabilities) where priorities differed in emphasis between those affected by the condition and those who study the condition [16]. By incorporating the expertise of the people most affected by preterm birth at all levels of the research enterprise and centering their priorities in development of a research agenda, we believe that the research produced will be more effective and more likely to lead to greater health equity.

### Local Application of the Priority Topics and Questions in Research Strategy and Funding Decisions

Over the course of the project, PTBi-CA incorporated the reseach priorities of women in Fresno, Oakland and San Francisco in its research strategy and funding decisions. We first invited women who participated in this project, and from other partnerships within their community, to join a Community Advisory Board (CAB) to advise PTBi-CA on the research strategy and conduct of research. We incorporated the different priorities from this project into biannual calls for proposals. The CAB contributed to a revision of the standard university research application to include plain English summaries and greater emphasis on community partnership and research dissemination plan. The CAB reviews all research proposals and participates in funding decisions. The CAB also regularly interacts with active and potential investigators to increase community partnership literacy among researchers and research literacy among community members. Over the course of the initiative, we have increased the number of projects with community Co-Principal Investigators, Co-Investigators and Consultants. Several of the CAB members or community research partners have transitioned to employment in a variety of research roles. Some of our earlier research grantees have gone on to include community partnership in other research studies, not funded by PTBi-CA. We have attempted to form authentic university partnership with communities by investing in existing infrastructure through funds flow to trusted community-based organizations, rather than create or co-opt infrastructure for the university. This is important for keeping the locus of power and control as close to the community of origin as possible. Finally, we have worked to disseminate the findings of the work in partnership with the individuals, groups and communities involved in the work, by providing community report-backs, co-presenting at professional meetings and co-authoring posters and academic papers with participants and community partners. We also have made the questions and topics generated publicly available so that research funders, researchers and communities can pursue or advocate for research on these priorities (https://pretermbirthca.ucsf.edu/developing-research-strategy-partnership-communities-affected-preterm-birth).

### Lessons learned

An unexpected positive outcome from this work was the interest expressed and further engagement from many participants in PTBi-CA research and related activities. Not all participants became actively involved in preterm birth research, yet the experience of participating in RPAC sessions clearly awakened curiosity, interest aspiration as indicated by the evaluation comments of many of the participants.

During the conduct of this work, we found that the RPAC method was very effective for developing a research agenda that includes the priorities of those most affected by the condition in question, and as a recruitment tool for deepening partnership in research. We also discovered that those commissioning the work must have a clear plan for how the research priorities will inform research strategy and funding decisions. Failure to do this will compromise credibility between the research funders or researchers and the community, and adversely affect future partnership. We also learned that it is important to work with and through community-based organizations who are trusted and well-integrated into the community of interest and that sufficient resources must be allocated for this. The community-based organizations and group facilitators must be prepared for discussions about personal and historical misconduct in research because this will likely be raised as a concern by potential or actual participants. Others have noted the importance of respect, equitable powere and trust as well as training, financial compensation and regular dialogue in successful patient stakeholder – researcher partnerships [40].

### Implications for National Research Funding Priorities

Conducting research within a Research Justice framework, means not only recognizing the expertise of communities of color, indigenous peoples and other marginalized groups, but also valuing that these groups have power in creating knowledge, and most importantly, in decisions about what research is conducted. In 2016, an estimated $171.8 billion was spent in the US on medical and health research and development, of which 67.5% was funded by industry [41]. It is not known how much of this spending was devoted to topics related to pregnancy, birth and newborn health conditions, or to topics related to interpersonal or structural health inequities. However, it is likely that even a small shift in the US maternal child health research agenda [42] toward the priority questions and topics generated by the participants in this project could result in a shift to greater proportion of investment in health service delivery, social determinates of health, or policy research as compared with basic or translational research. It also most certainly would require changes in the focus and conduct of industry-funded research. These are challenging questions that disrupt existing power structures, which may not be easy to shift. However, communities most affected by health disparities are reclaiming and wielding research knowledge and power and developing new structural norms for the research enterprise that may offer a way forward. The Black Mamas Matter Alliance, for example, has just released a landmark report that details recommendations for conducting research “with, for and by Black Mamas” [43]. Another group that provides guidance on addressing the main inequities that exist in the research enterprise is Chicago Beyond. Their 2018 report [44] provides a detailed guidance for community organizers, researchers and research funders to disrupt the traditional power dynamics in the research enterprise so as to create higher quality evidence to improve health and equity. Specific to healthcare research, the Institute for Patient and Family Centered Care (IPFCC) recently published recommendations for increasing diversity in patient and community partnership in research [45]. By adopting a Research Justice framework, and recommendations of groups such as the Black Mammas Matter Alliance, Chicago Beyond and IPFCC, research funders, research institutions and researchers can more fully partner with communities that bear the burden of health disparities and may achieve a greater return on investment for both research and for society.

### Limitations

The methods and results of this work must be considered in light of the limitations. First, the questions generated by the participants should be considered as potential research questions and, like any initial idea for research, need to be considerably refined after a thorough review of the literature and consulation with experts on potential research designed. Nevertheless, it is interesting to note that there were very few questions posed by the participants that had definitive evidence such that no further research is needed. This underscores the dearth of evidence that persists in the field of pregnancy and childbirth. Second, questions and topics generated by the groups in this study are not necessarily representative of all women of color at risk for preterm birth, nor are the research priorities of these women likely to remain static over time. The state of knowledge and experiences is dynamic and ever-changing. We cannot at this stage in the development of the RPAC protocol make recommendations about how many groups to convene, or how often to repeat the process. However, we do know that communities who have experienced health disparities and research injustice are extremely sensitive to inauthentic engagement with researchers. They want to see that the investment they have made in any partnership results in action that benefits the community. Therefore, it is important that there is a clearly defined pathway to research on at least some of the questions or topics generated and that action is well-communicated before re-engaging to solicit more priorities. Moreover, it may be that with greater and deeper partnership, communities will pro-actively communicate with funders and researchers if priorities need to be shifted or updated.

Third, during the course of this work we noted opportunities within the RPAC protocol to deepen community voice. For example, with additional time, participants can be involved in the thematic analysis of the questions generated and create the topic labels, instead of the research team creating and validating them with participants. We also noted that in some groups, participants had more difficulty ranking topics than specific questions. This was because we did not have a robust process for decision-making, other than by majority vote, when there were choices of rank to be made and lack of group consensus. We noted individuals acquiescing to others in the group based on empathy and reinforcement from the facilitators that all topics (or questions) were important and that the rankings only indicated priority for urgency to begin. It was important to both acknowledge this dynamic and ensure that participants felt their questions were reflected in the final priorities. Unfortunately, communities that experience the greatest burden of health inequities have not been adequately resourced to develop a shared agenda toward health equity [46] and we witnessed an internalization by participants of the notion that there are few resources to be dedicated to their priorities, rather than a more expansive view that more resources could be dedicated to their priorities. We also noted limitations in regard to generating a priority list across regions. We utilized a post-hoc weighted ranking method, but this may not represent consensus or the majority preferences. Among the possible improvements to this process could include putting all of the generated questions in a Delphi-type survey [47] to the cumulative participants, or to their community at large, or if more than one group is planned.

Fourth, robust methods do not currently exist to measure and monitor the impact of RPAC or any of the other methods for community partnership in research agenda setting or resource allocation, other than within the narrow frame of an individual funding body [48]. Moreover, some have called into question whether patient and public involvement in research does indeed result in better quality evidence or translation to practice [18]. PTBi-CA can precisely track the ways in which our use of the RPAC protocol has impacted the research we conduct or commission, how that research is conducted and, to a limited extent, how that research is translated into practice and future research. However, to our knowledge, there are no measures at present of how giving voice to the unanswered questions and priorities of affected communities influences the larger national research investments of government and industry. There currently exist no readily available analyses of medical and health research funding by topic area or population, nor whether the funding decisions included affected communities.

### Future directions

The field of community partnered research strategy setting is still quite immature and there are many opportunities to advance knowledge and capacity. We specifically refer readers to the growing literature in this field, inclusive of but not limited to the examples cited in this paper, and encourage application and comparison studies to identify criteria for the selection of the best approach and comparative effectiveness. However, the latter requires further work to develop a set of outcome metrics and measures, which are currently lacking. Innovations are also needed to develop methods for generating research priorities of affected communities that can accommodate larger samples, without losing the quality or context of the question as is captured in the RPAC focus group method, for example.

Despite the great need for further research, the findings from this work can be directly applied in practice. Healthcare and social service providers can use the available evidence to improve services related to the community’s priority topics and questions. It was clear from the discussions in all of the groups that patients/clients and families notice, and are concerned by, the inconsistent practices of providers and institutions. When the inconsistencies are coupled with, often contradictory, statements about evidence, it erodes community trust in healthcare providers and the system. Health and social care providers can educate patients/clients and families on what is already known about the priority topics. This can increase health literacy, empowerment, trust and service quality [2, 49]. Finally, health and social care providers can immediately work to improve the practice of respectful care and shared decision-making in all patient/client interactions. Although further research is needed on more effective ways to build and sustain a culture of respect and shared decision-making, the findings from this work indicate deep community concerns about individual and structural problems leading to disrespectful and paternalistic care that can be addressed immediately without waiting for further research [50].

Research funders and researchers can use the findings from this work to focus resources to address the unanswered questions that matter most to women at high risk for preterm birth about pregnancy birth and newborn health. Next steps include collating or updating systematic literature reviews to further define the research gaps, and then to commission (or propose) studies to address these questions. This may include development of new interventions or research methods where prior research on the topic has been under-developed, for example, in interventions research to reduce personal and work stress or interpersonal and structural racism during pregnancy and the post-partum period. Research funders and researchers can, and must, apply the available methods to work in partnership with women, families and communities most affected by the preterm birth epidemic in research agenda setting and in conducting research, while continuing to innovate and advance the methods.

## Conclusions

In summary, we encourage research funders and researchers to work in partnership with communities at high risk for preterm birth and to use the findings from this work to focus resources to address the unanswered questions that matter most to women at high risk for preterm birth. We also encourage continued innovation to advance the methods of research priority setting with affected communities. We encourage all researchers and research funders to endorse and fully adopt a Research Justice framework and to conduct research according to the BMMA essential components for conducting research: 1) Communities have a right to be recognized and own authoritative community expertise; 2) Communities have a right to know; and 3) Communities have a right to be heard [43].

## Data Availability

Study data, methods and training video available from: https://pretermbirthca.ucsf.edu/developing-research-strategy-partnership-communities-affected-preterm-birth

## Appendix 1

**Table:** Weighted Mean Rank of Priority Research Topics by Region

Fresno	Oakland	San Francisco
1. Stress and the Benefits of Social Support	1. Health Care Providers	1. Medications, Procedures and Tests During Pregnancy
2. Mental Health Support	1. Mother’s Health Before and During Pregnancy^1^	2. Care Provision During Pregnancy and Birth
3. Newborn Health and Problems Related to Prematurity	2. Newborn Health and Problems Related to Prematurity	3. Stress and the Benefits of Social Support
4. Medications, Procedures and Tests During Pregnancy	2. Stress and the Benefits of Social Support^1^	4. Newborn Health and Problems Related to Prematurity
4. Mother’s Health Before and During Pregnancy^1^	3. Medications, Procedures and Tests During Pregnancy	4. Care After Birth^1^
5. Hospital and Health System Practices	4. Decision Making During Pregnancy	4. Communication and Cultural Sensitivity
6. Care After Birth	5. Education and Empowerment of Birthing Women	5. Workplace and Insurance Issues
7. Education and Empowerment of Birthing Women	5. Maternal Nutrition	6. Health Care Providers
7. Support for Fathers During and After Pregnancy^1^	6. Infant Nutrition, Feeding and Medications	Mother’s Health Before and During Pregnancy^2^
7. Workplace and Insurance Issues	7. Care After Birth	Education and Empowerment of Birthing Women^2^
8. Role of Family and Friends in Caring for Families	8. Hospital and Health System Practices	Infant Nutrition, Feeding and Medications^2^
9. Care Provision During Pregnancy and Birth	8. Care Provision During Pregnancy and Birth	
10. The Impact of Men on Pregnancy Outcomes	9. Innovations in Care Models	
11. Health Care Providers	10. Communication and Cultural Sensitivity	
12. Communication and Cultural Sensitivity	11. Workplace and Insurance Issues	
13. Preconception, Contraception and Sexuality	12. Preconception, Contraception and Sexuality	
14. Pharmaceutical Company Involvement in Birth	13. Support for Fathers During and After Pregnancy	
15. Decision Making During Pregnancy	14. The Impact of Men on Pregnancy Outcomes	
16. Infant Nutrition, Feeding and Medications		

^1^Tied rank with prior topic

^2^Unranked topics

## Appendix 2

**Table:** Top Priority Potential Research Questions By Group

Rank	Fresno - Black Infant Health	Fresno - Every Neighborhood Partnership	Fresno – Spanish only group	Oakland - Best Babies Zone	Oakland - Black Wellness Zone	San Francisco - Black Infant Health	San Francisco - Homeless Prenatal Program
1	Why aren’t there more widely available education materials or videos, etc. to help women recognize their own stress?	Is it harmful to babies to be separated from mom (family) even if they are sick?	Why do we have unpleasant experiences at the hospital?	How can women learn about pregnancy risk factors before pregnancy?	Why don’t we have alternative options for support during delivery? (For example, doula, midwife)	Why are nurses disrespectful and/or do not believe patients? How do negative attitudes among nurses develop? Can they be improved? What are the most effective ways to improve patient-provider communication, particularly when patients perceive insensitive and rude comments from health care workers? How can women report insensitive and rude health care worker communication? How seriously are complaints heard?	How does a mother’s stress affect the baby? Does stress cause a preterm delivery? What is the impact of stress on preterm birth and birth?
2	Why aren’t we given tools to reduce stress?	How can ER + ambulance staff be better prepared for preterm babies-equipment and training? Including progress, outcomes and understanding families.	Is there a connection between autism and certain medications?	What are the factors that determine how staff interact with patients?	What extra care do preemies need?	If AA women are at higher risk, why isn’t there specialized care to improve outcomes?	Why is pregnancy care different depending on where you get care? What is the most effective care for pregnancy and high risk pregnancy? How different is the care at different locations and why?
3	How can emergency room and ambulance staff be better prepared for preterm babies-equipment and training?	What testing should be done to find blood conditions during pregnancy?	If I’ve had a preterm birth in the past, will I have a preterm birth in the future?	How accurate is genetic testing? When should it be done?	Why do some women make more milk and others don’t?	What causes SIDS?	Is care different based on insurance status or race? Does the type of insurance you have determine the type of care that you get? Or the quality of care?
4	What mental supports should parents seek after PTB/Birth trauma?	What can women do when they have concerns that are not listened to?	Why are we not seen promptly in an emergency room if we are having an emergency?	How is high risk decided? How are women told about it?	So what if MD can’t explain your illness or your condition to you, what are they supposed to do?	What workplace supports are effective to prevent preterm birth? How does stressful physical work affect pregnancy?	What is the evidence for safety of medicines during pregnancy? What is the impact of fentanyl and other medications during pregnancy and breastfeeding?
5	Why aren’t doulas offered (free/covered by insurance)?	What can women do when they disagree with the medical advice?	Why are doctors not more careful regarding the medications they are prescribing to us during pregnancy?	Are there health risks associated with pregnancy?	Why is age a risk factor for problems?	What’s the best diet for a breastfeeding mom so the breast milk has the most nutrition?	What is the role of Child Protective Services (CPS) and reporting of women during pregnancy care? How do health care providers decide to involve CPS during pregnancy care when abuse and neglect are not clearly present? How do they pick and choose family?
6	Why aren’t there standard pregnancy services the same for all types of insurance	What mental supports should parents seek after PTB, birth trauma?	I still have pain after being administered my epidural over a year ago. Why is this?	Why don’t providers tell you after effects of getting your tubes tied?	How can staff provide better communication?	How can the health care team better follow and read the birth plan? Why isn’t review of birth plans part of the providers’ script? How do birth plans help?	Could train-the-trainer experienced women moms be used more effectively for breast feeding support? (HPP) Why does breast milk dry up? Is pumping and feeding as good as breastfeeding? Should babies be put to breast more than pumping? Study breast milk production.
7	Why is BIH the only Culturally-centered pregnancy support service in Fresno?	What kind of follow up and home support can be provided to help parents “take care of their babies at home and” make sure they were given right treatments or know signs of problems?	Why did I still feel pain despite having been administered an epidural?	Is it normal the doctors tell you that you have to have a C-section?	What are the holistic ways I can reduce stress during pregnancy-with work and family stress?	Is care different based on insurance status or race? Does the type of insurance you have determine the type of care that you get? Or the quality of care?	Why do babies receive morphine medications and fluids and what are the risks? Why is it desirable to not allow babies to move? Why does the NICU team not want babies to move around?
8	What causes pregnancy loss?	What support do men need during pregnancy and in the NICU?	Why do medical providers hurt us so much when performing physical exams on us during pregnancy and when we come in for our ultrasounds?	Why do doctors talk down to patients?	Why do a very few women get pregnant while on birth control?	Could train-the-trainer experienced women be used more effectively for breast feeding support? (experienced moms)	What are the most effective ways to improve patient-provider communication, particularly when patients perceive insensitive and rude comments from health care workers? How can women report insensitive and rude health care worker communication? How seriously are complaints heard?
9	Why are (n’t) more options for birth? (water birth)?	Is there risk to extended family bonding in the NICU?	Is there something that we can do as mothers to help our babies when they are in the NICU?	How real is confidentiality? (between physicians and others)	Why would I have a spontaneous pneumothorax? No known risk factors.	What are the best “home remedies” for babies and preemies? What are the best at-home formula preps?	Does a mother’s age contribute to preterm age?
10	What can friends and families do to support families in the NICU?	Why does it take so long doctors to decide to do further diagnostic testing after multiple miscarriages?	What level of bleeding is normal during pregnancy?	Can the father’s affect, effect the women’s state of pregnancy?	When babies smile at young newborn age is it gas or something else? Such as “antho” kicking in spirits, ancestors thoughts?	Is marijuana safe during pregnancy? Is it safe/is it beneficial for the baby?	What supports are most helpful for moms with children at home (with no support)? What could make hospital visits and in-hospital stays easier for families?
11	Why do I have more than one doctor?	What causes placental abruption?	Which medicines have both a negative and positive impact on pregnancy?	Do men affect a woman’s risk during pregnancy blood type, DNA?	Do you have the option to change doctors and nurses?		How do decisions about discharge get made between families, insurance companies, providers, parents? What are the best practices (difference hospital stay)?
12				Why don’t physicians explain what they are doing and when you will see them?	What more can be done to allow moms to have skin-to-skin /BF after anesthesia?		How do needed dental procedures during pregnancy affect the baby?
13				Why do we get conflicting advice about diet?	What controls weigh gain during pregnancy?		What are the best ways to establish trust between pregnant women and care givers? What are effective strategies to build trust with patients?
14				Why do you have to give birth on your back?			What are the best strategies for emotional support and preparation for parents at risk for preterm parenthood?
15							Why are nurses disrespectful and/or do not believe patients? How do negative attitudes among nurses develop? Can they be improved?

## Abbreviations

BMMA: Black Mamas Matter Alliance
CAB: Community Advisory Board
CBO: Community Based Organization
CBPR: Community Based Participatory Research
CPS: Child Protective Services
HELLP: Hemolysis, Elevated Liver enzyme levels, and Low Platelet
IPFCC: Institute for Patient and Family Centered Care
JLA: James Lind Alliance
NICU: Neonatal Intensive Care Unit
PPI: Patient and Public Involvement
RPAC: Research Priorities of Affected Communties
SIDS: Sudden Infant Death Syndrome
US: United States
